# The Emerging Role of CYP17A1 in Diabetes and Related Metabolic Disorders

**DOI:** 10.3390/ijms27219826

**Published:** 2026-11-02

**Authors:** Zainab Alhalwachi, Mashael Zainalabedin, Daleen Alhayki, Abdullah Buraq Alsultan, Siham Abdullahi, Kawther Ahmed Alnahwi, Alexandra E. Butler, Ammar Shaker Hasan

**Affiliations:** 1 Department of Postgraduate Studies and Research, Royal College of Surgeons in Ireland, Medical University of Bahrain Busaiteen, Adliya P.O. Box 15503, Bahrain; zalhalwachi@rcsi-mub.com (Z.A.); mzainalabedin@rcsi-mub.com (M.Z.); dalhayki@rcsi-mub.com (D.A.); abutler@rcsi.com (A.E.B.); 2 Department of Medicine, Royal College of Surgeons in Ireland, Medical University of Bahrain Busaiteen, Adliya P.O. Box 15503, Bahrain; 3 College of Medicine, Alfaisal University, P.O. Box 50927, Riyadh 11533, Saudi Arabia

**Keywords:** CYP17A1, androgen, steroidogenesis, metabolism, type 2 diabetes

## Abstract

CYP17A1 (cytochrome P450 17A1) is a key steroidogenic enzyme that catalyzes 17α-hydroxylase and 17,20-lyase reactions, controlling glucocorticoid and androgen production. Emerging evidence suggests that CYP17A1 is not only essential for adrenal and gonadal steroidogenesis, but could also contribute to obesity, insulin resistance, type 2 diabetes, and hypertension. Genetic studies have associated CYP17A1 variants with fasting glucose, type 2 diabetes risk, arterial stiffness, and metabolic syndrome traits. In mouse models, obesity induces hepatic CYP17A1 expression and 17-hydroxyprogesterone overproduction, which activates glucocorticoid receptor signaling, promotes hepatic gluconeogenesis, and exacerbates insulin resistance and hyperglycemia. CYP17A1 has also been linked to adipose tissue dysfunction, altered steroid signaling, and redistribution of fat from visceral to subcutaneous depots. This review summarizes CYP17A1 structure, regulation, protein interaction networks, and its roles in insulin resistance, adipose tissue biology, steroid excess, and vascular complications. It also discusses CYP17A1 genetic variation and the therapeutic potential and limitations of CYP17A1 inhibition and glucocorticoid receptor modulation in diabetes and related metabolic disorders.

## 1. Introduction

Diabetes Mellitus (DM) presents as a significant global health burden in the 21st century, with the International Diabetes Federation (IDF) reporting that, in 2024, the worldwide prevalence of diabetes among adults aged 20–79 years was estimated to be approximately 589 million (11.1%) [1]. Type 2 diabetes mellitus (T2DM) is the most common form of diabetes, accounting for more than 95% of cases worldwide [2], and is characterized by hyperglycemia and β-cell dysfunction, after the presence of obesity, insulin resistance (IR), and metabolic syndrome (MS) [3,4]. Long-term complications of T2DM include non-alcoholic fatty liver disease, coronary heart disease, stroke, diabetic kidney disease, retinopathy, as well as an increased risk of developing dementia and specific cancers [5]. Therefore, it is not surprising that T2DM patients have a reduced life expectancy of 7.67 years compared to non-diabetics of the same age group [6] and that T2DM is the ninth leading cause of mortality worldwide, with a toll of ≥1 million deaths annually [7].

Despite the growing awareness of T2DM complications, and the various available treatments for the chronic metabolic disorder, ranging from lifestyle intervention, oral medication and insulin therapy [8,9], the IDF projects that global diabetes prevalence will rise to approximately 853 million adults by 2050 [1]. T2DM is a complex multi-system and multi-organ disorder, arising from defects across a multitude of metabolic, hormonal, genetic, and inflammatory pathways [10]. This results in heterogeneity between T2DM patients, and thus, it is difficult to develop a singular therapy that can ubiquitously treat all diabetic patients [11]. Furthermore, many presently available treatments for T2DM lack long-term efficacy, as they do not address the complex metabolic dysregulation that leads to progressive islet β-cell dysfunction [12]. As such, it is evident that current treatments and intervention plans are not adequately meeting the T2DM global health burden; hence, elucidating new therapeutic targets for the metabolic disorder is imperative to combat the rising incidence and mortality rates.

Human steroidogenic P450 (CYP) enzymes are being increasingly explored as targets for metabolic diseases. These enzymes maintain essential basal physiological homeostasis in the body through steroid and hormone biosynthesis, as well as metabolism of fatty acids. Therefore, disruptions in their enzymatic activity lead to complex metabolic dysregulation, such as Cushing’s syndrome [13]. Additionally, variants in the CYP11A1, CYP17A1, and CYP19A1 enzymes have been associated with metabolic diseases including polyendocrine metabolic ovarian syndrome (PMOS), formally polycystic ovary syndrome (PCOS), and T2DM [14]. With research linking the dysregulation of CYP enzymatic function to various metabolic disorders, exploring these proteins as therapeutic targets for conditions that clinically present complex metabolic disruption, like diabetes, is of growing interest.

CYP17A1 is a promising yet underexplored potential target for T2DM. This protein is the key steroidogenic enzyme involved in glucocorticoid (GC) biosynthesis [15]. GCs, like cortisol, regulate multiple facets of glucose homeostasis, including hepatic gluconeogenesis, glycogen metabolism, as well as the glucagon and insulin secretion functions of pancreatic α and β cells [16]. When GC levels are chronically elevated due to metabolic dysregulation, excess GC exposure leads to the development of hyperglycemia, insulin resistance, and ultimately, diabetes [17]. Thus, exploring the role of CYP17A1 in excess GC biosynthesis can elucidate candidate mechanisms that could be utilized to treat T2DM. Moreover, CYP17A1 presents as a potential target to address whole-body metabolic dysregulation, as studies have showcased gene expression changes in lipid, glucose, and insulin signaling in Cyp17a1-knockout rats, as well as altered adipose tissue metabolism [18]. Therefore, it is evident that CYP17A1 inhibition should be further researched as a potential mechanism to combat metabolic dysregulation in diabetic patients. Overall, this review aims to explore the structures, roles, and impact of CYP17A1, uniquely in the narrative of T2DM and other related metabolic disorders. By highlighting key findings on the enzyme, this review strives to advance therapeutic treatments involving CYP17A1, which can address current pitfalls in T2DM treatments that fail to address metabolic dysfunction.

## 2. Structure and Function of CYP17A1, the Steroidogenic Enzyme

### 2.1. The Structure of CYP17A1, the Steroidogenic Enzyme

Cytochrome P450 17A1 (CYP17A1) is a ~57 kDa microsomal enzyme made up of ~508 amino acids [19]. It is encoded by a gene spanning approximately 6.6 kb on chromosome 10q24.3, and contains 8 exons and 7 introns. Translation from a single 2.1 kb mRNA species occurs in the adrenals and gonads [20,21]. It is anchored in the endoplasmic reticulum, containing four important domains: substrate binding domain, a hydrophobic region on the distal end of the globular catalytic domain, a heme-binding region and a redox-partner binding site [22]. CYP17A1 is a crucial contributor to steroidogenesis, as it catalyzes both 17α hydroxylase and 17,20 lyase reactions, which modulate glucocorticoid and androgen production fundamental for systemic metabolic homeostasis.

### 2.2. Protein-Protein Networks

**Figure 1:**
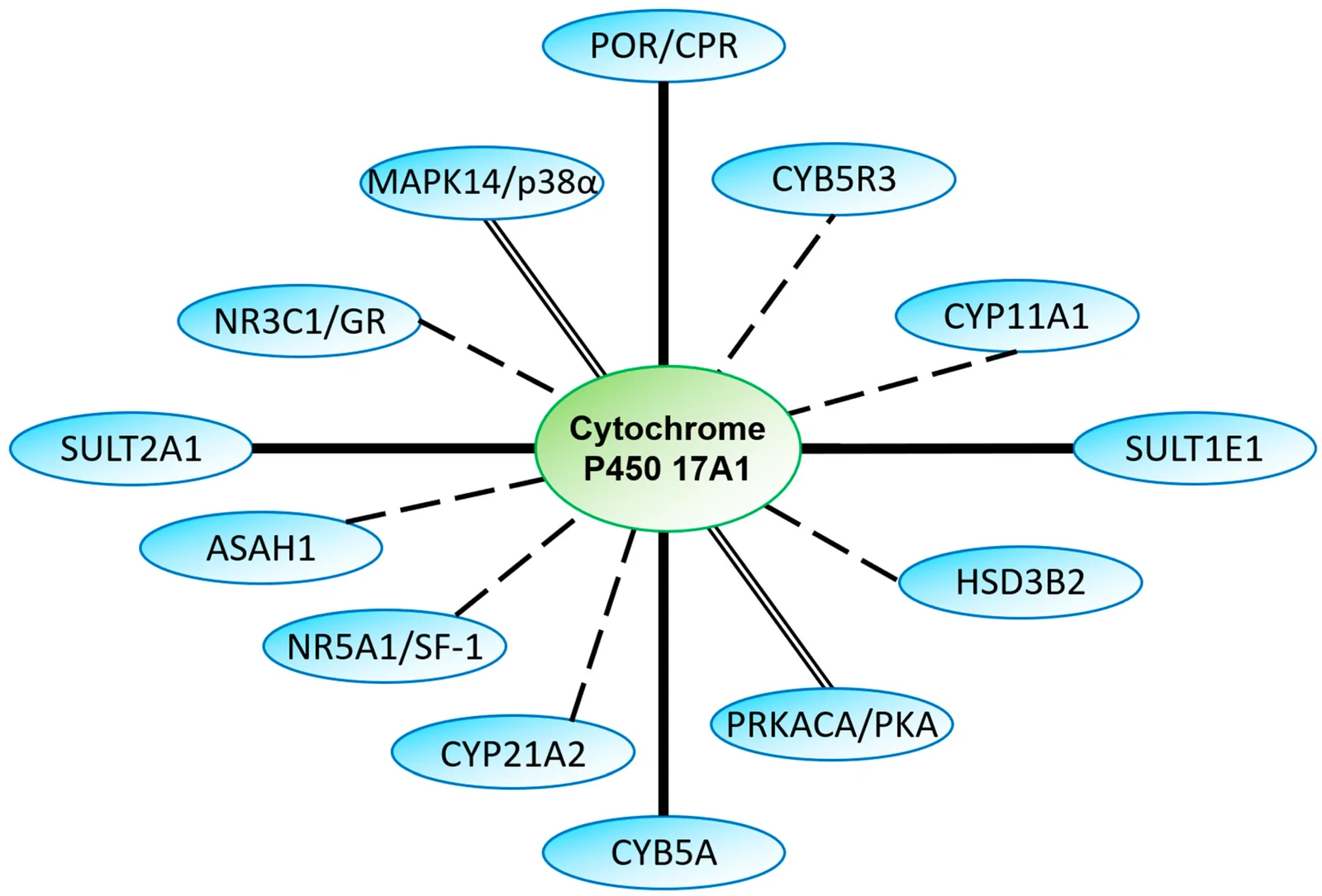
Literature-derived protein-interaction network of cytochrome P450 17A1 (CYP17A1). The network was constructed manually using a narrative review of published literature. Nodes represent CYP17A1 and proteins identified in reviewed literature as having pathway associations, direct protein or functional interactions. Edges are classified according to experimental evidence: Thick solid lines indicate confirmed direct protein–protein interactions, double lines represent direct functional interactions, and dotted lines denote indirect or pathway-associated interactions. Abbreviations: CYP17A1, cytochrome P450 family 17 subfamily A member 1; POR, cytochrome P450 oxidoreductase, also known as NADPH–cytochrome P450 reductase; MAPK14, mitogen-activated protein kinase 14 (p38α); CYB5A, cytochrome b5 type A; SULT1E1, sulfotransferase family 1E member 1; HSD3B2, 3β-hydroxysteroid dehydrogenase type 2; PRKACA, protein kinase cAMP-activated catalytic subunit α (protein kinase A catalytic subunit); CYB5R3, cytochrome b5 reductase 3; CYP11A1, cytochrome P450 family 11 subfamily A member 1; CYP21A2, cytochrome P450 family 21 subfamily A member 2; NR3C1, nuclear receptor subfamily 3 group C member 1 (glucocorticoid receptor); SULT2A1, sulfotransferase family 2A member 1; ASAH1, N-acylsphingosine amidohydrolase 1 (acid ceramidase); NR5A1, nuclear receptor subfamily 5 group A member 1 (steroidogenic factor 1).

The CYP17A1 protein–protein interaction network includes partners that differ in the strength and nature of their evidence, where the following is summarized in Figure 1. P450 oxidoreductase (cytochrome P450 reductase) (POR/CPR) is a confirmed direct redox partner that transfers electrons from nicotinamide adenine dinucleotide phosphate (NADPH) to CYP17A1, supporting both 17α-hydroxylase and 17,20-lyase activities. Cytochrome b5 type A (CYB5A) is also a confirmed direct interactor; it binds the proximal surface of CYP17A1 and allosterically promotes the interaction between CYP17A1 and POR, thereby selectively enhancing 17,20-lyase activity. Important residues contributing to the CYP17A1–CYB5A interface include CYP17A1 Arg347, Arg358, and Arg449 and acidic residues Glu48 and Glu49 of CYB5A [23,24].

Sulfotransferase family 1E member 1 and sulfotransferase family 2A member 1 (SULT1E1 and SULT2A1) are additional direct CYP17A1 interactors. Surface plasmon resonance experiments demonstrated direct binding of CYP17A1 to SULT1E1 and SULT2A1, with reported dissociation constants of approximately 196 nM and 124 nM, respectively. These interactions support a proposed multienzyme complex involved in the coordinated synthesis and sulfation of steroid products, including dehydroepiandrosterone (DHEA) and DHEA sulfate [25].

Mitogen-activated protein kinase 14 (p38 alpha) (MAPK14/p38α) and protein kinase cyclic adenosine monophosphate (cAMP)-activated catalytic subunit alpha/protein kinase A (PRKACA/PKA) are classified as direct functional interactions because they regulate CYP17A1 through phosphorylation rather than serving as constitutive redox or metabolic complex partners. MAPK14/p38α physically associates with CYP17A1 in co-immunoprecipitation experiments and phosphorylates the enzyme at a noncanonical site, selectively increasing 17,20-lyase activity and androgen biosynthesis. PKA also phosphorylates human CYP17A1, particularly at Ser258, and this modification increases CYP17A1 catalytic activity [26,27].

The remaining proteins in the network represent indirect or pathway-associated relationships. Cytochrome b5 reductase 3 (CYB5R3) may support CYP17A1 function indirectly by maintaining CYB5A in its reduced state, but direct binding between CYB5R3 and CYP17A1 has not been established. CYP11A1, 3β-hydroxysteroid dehydrogenase type 2 (HSD3B2), and CYP21A2 are sequential or competing steroidogenic enzymes that influence the availability and metabolic fate of CYP17A1 substrates, rather than confirmed physical binding partners. NR5A1/Steroidogenic Factor-1 (SF-1) regulates CYP17A1 transcription by binding its promoter, whereas N-acylsphingosine amidohydrolase 1 (acid ceramidase) (ASAH1) regulates CYP17A1 indirectly through its interaction with SF-1. Finally, nuclear receptor subfamily 3 group C member 1/Glucocorticoid Receptor (NR3C1/GR) acts downstream of CYP17A1-derived steroid products, particularly 17-hydroxyprogesterone, and does not directly bind the CYP17A1 enzyme [28,29,30,31].

### 2.3. The Function, Pathways and Regulations of CYP17A1 in Steroidogenesis

CYP17A1 is important in the regulation and production of steroids in the steroidogenic pathway [32]. The enzyme’s functions, pathways and regulations have been elucidated over time through various *in vitro* and *in vivo* studies across different species. In the human adrenal gland, following the conversion of cholesterol to pregnenolone by CYP11A1 [33], pregnenolone is either converted to progesterone (Δ4 pathway) or 17-hydroxypregnenolone (17-OHPreg) (Δ5 pathway). The direction of the pathway is dependent on site-specific differential enzyme expression within the adrenal tissue, which is composed of three layers: zona glomerulosa, zona fasciculata, and zona reticularis. Each layer expresses different levels of key enzymes, including CYP17A1 and HSD3B2, that determine the direction of steroidogenesis [34].

Trophic protein hormones control the expression of CYP17A1 within humans in a tissue- and zone-specific manner. Adrenocorticotropic hormone (ACTH) stimulates CYP17A1 expression in the zona fasciculata and zona reticularis via the cAMP/protein kinase A (cAMP/PKA) signaling cascade. As a result, there is an enhancement of transcription of steroidogenic enzymes involved in glucocorticoid and adrenal androgen biosynthesis [35,36,37]. Conversely, the zona glomerulosa does not express CYP17A1; regulation is controlled by AngII-induced signals and Wnt/β-catenin activation, ultimately producing aldosterone [37].

In contrast, the expression of CYP17A1 in gonadal tissue is regulated by luteinizing hormone (LH) via a similar cAMP/PKA signaling cascade. It has been shown in rodent ovarian theca cells and testicular Leydig cells that LH promotes androgen biosynthesis by activating SF-1 and Nur77 (nuclear receptors), thereby sustaining androgen production required for follicular steroidogenesis [36,38]. However, the outcome of this depends on cellular differentiation. During luteinization in pre-ovulatory granulosa cells, CYP17A1 is markedly repressed via induction of the transcriptional repressor KLF4, leading to progesterone accumulation [39]. Yet, LH in male gonads maintains a stimulatory role in rodent Leydig cells, sustaining the activation of transcription factors that support testosterone production. Additionally, inflammatory cytokines like TNF-α trigger NF-κB signaling, which inhibits the activity of SF-1 and Nur77, and can thus disrupt the responsiveness of Leydig cells to LH, leading to reduced CYP17A1 expression [38]. However, these findings are primarily based on rodent studies and require confirmation in human Leydig cells, to confirm that CYP17A1 regulation reflects integration of endocrine, transcriptional, and inflammatory signaling inputs in humans too.

CYP17A1 expression has also been shown to be controlled by metabolic and inflammatory pathways across species. Insulin-like growth factors IGF-1 and IGF-2 upregulate CYP17A1 transcription and enhance both hydroxylase and lyase activities, by activating the IGF-1 receptor and the Phosphatidylinositol 3-kinase (PI3K) signaling cascade, which links metabolic inputs to steroidogenic output in yellowtail fish ovarian follicles [40]. Alternatively, pro-inflammatory cytokines such as TNFα, IL-1β, and IL-6 suppress androgen production in both theca and Leydig rat cells by downregulating CYP17A1 expression [41]. The regulation of CYP17A1 also extends beyond transcription in humans; it also involves post-translational modulation, which is mediated by estrogen-induced microRNA pathways [42]. In summary, these mechanisms demonstrate that CYP17A1 expression is controlled by an intricate network of hormonal, metabolic, inflammatory, and post-transcriptional controls across various species that warrant further investigation.

### 2.4. The Effect of Metabolic and Endocrine Stress on CYP17A1

Metabolic and endocrine stressors like obesity, insulin resistance, and chronic inflammation are known to alter steroidogenic pathways. As CYP17A1 plays a pivotal role in glucocorticoid and androgen biosynthesis, disruptions in endocrine signaling pathways may lead to downstream effects on steroidogenesis, which in turn manifest as systemic metabolic dysfunction. Obesity is associated with a chronic state of stress and has been found to alter the balance between glucocorticoids and androgen production [43]. This is due to the extensive expansion of adipose tissue, which results in the secretion of pro-inflammatory cytokines and free fatty acids, leading to low-grade inflammation [44].

Additionally, this chronic state of stress disrupts the Hypothalamic–Pituitary–Adrenal (HPA) axis in the body and alters cortisol and dehydroepiandrosterone DHEA production, the latter being the naturally occurring precursor to major sex hormones like testosterone and estrogen. Increasing the cortisol-to-DHEA ratio impacts steroidogenesis, and thus, obesity modifies CYP17A1-dependent pathways [45]. For instance, a study examining CYP gene expression in metabolic syndrome found that obesity-related metabolic stress alters steroidogenesis, with downregulation of CYP17A1 in these states [46].

Moreover, another study assessing how inflammatory signaling influences adrenal gland steroid hormone production demonstrated that during inflammatory stress states, IL-6 and TNF-α alter adrenal hormone production by interfering with enzymes involved in androgen production, i.e., CYP17A1, thereby upregulating the enzyme [47,48]. In metabolic disorders, inflammatory signaling pathways activate transcription factors such as NF-κB, which interfere with hormonal signaling pathways and contribute to excess androgens. This is more evident in PMOS, a metabolic disorder [48]. Therefore, it can be suggested that obesity-related metabolic stress and chronic inflammation can affect CYP17A1 function in the pituitary, adrenal glands, and adipose tissue.

## 3. Diabetes and Metabolic Disorders

### 3.1. Gluconeogenesis and Insulin Resistance

Glucose, via gluconeogenesis and glycogenolysis, is secreted from the liver into the blood during the body’s fasted state to maintain euglycaemia. This process is highly controlled and occurs through the secretion of glucagon by pancreatic alpha-cells, which modulates the cAMP/PKA pathway. This then activates the gluconeogenic transcription factor, cAMP response element-binding protein (CREB), and the transcription of gluconeogenic genes, such as Forkhead box protein O1 (FOXO1), Phosphoenolpyruvate carboxykinase (PEPCK), and Glucose-6-phosphatase catalytic subunit 1 (G6PC), resulting in an increase in gluconeogenesis and an increase in hepatic glucose production (HGP) [49,50]. Similarly, glucocorticoids bind to glucocorticoid receptors (GRs) in the cytoplasm of hepatic cells, which indirectly leads to an increase in gluconeogenesis by enhancing the transcription of gluconeogenic genes such as PEPCK [51].

Insulin resistance is defined physiologically as an impaired response of tissues to insulin stimulation [52]. The actions of insulin are mediated by insulin receptors found on the cell surface [53]. Insulin resistance impacts different metabolic processes depending on the tissue. For instance, the liver plays a fundamental role in maintaining metabolic homeostasis by producing and storing glucose, synthesizing fatty acids, and distributing energy. In insulin-resistant states, there is an excess of glucose and fatty acids directed to the liver, leading to decreased proximal insulin signaling and ultimately, metabolic dysfunction, as insulin’s ability to suppress gluconeogenesis is decreased, increasing circulating glucose levels, which further propagates insulin resistance [52,54,55].

Conversely, skeletal muscle plays a key role in insulin-stimulated glucose disposal, thus, insulin resistance at this site significantly affects systemic glucose turnover. This is primarily due to impaired function of the glucose transporter GLUT-4 and defects in the insulin signaling cascade, including INSR, IRS1, PI3K, and AKT activity [52,54]. Finally, in adipose tissue, insulin suppresses lipolysis. When this tissue becomes insulin resistant, elevated free fatty acids and glycerol accumulate in the liver and skeletal muscle, where they further perpetuate persistent hyperglycemia [54,55]. This leads to the development of diabetes mellitus, which is diagnosed by fasting plasma glucose ≥ 7.0 mmol/L, or 2 h PG ≥ 11.1 mmol/L during the oral glucose tolerance test (OGTT), or HbA1C ≥ 6.5% (48 mmol/mol) [56].

### 3.2. Adipose Tissue Dysfunction and Steroid Signaling

Adipose tissue plays an essential role in maintaining metabolic homeostasis by regulating whole-body energy balance and glucose metabolism. It acts primarily as the energy reservoir, storing excess energy as neutral triglycerides (TGs) in adipocytes. In fasted states or increased energy expenditure, these TGs are broken down into fatty acids and glycerol and delivered to the muscle, liver, and other organs [57,58]. Adipose tissue also functions as an endocrine organ and secretes hormones and adipokines, which relay information to organs like the brain, muscle, and liver.

Although the exact mechanism that initiates adipose tissue dysfunction is not well understood, multiple intrinsic and extrinsic factors are presumed to contribute to its development. Amongst the intrinsic factors, chronic positive energy balance, increased nutrient influx into adipose tissue, and limited expansion of subcutaneous fat stores contribute to this dysfunction and can further lead to obesity. Extrinsic factors include a combination of genetic, environmental, and behavioral factors. This leads to the hallmark features of adipose tissue dysfunction: Adipocyte hypertrophy, ectopic fat deposition, and immune cell infiltration [58].

In obesity, adipocyte mitochondrial dysfunction reduces energy utilization, which in turn leads to adipocyte apoptosis, inflammation, and insulin resistance. Together, these factors promote metabolic dysregulation and further propagate insulin resistance [58]. Moreover, adipose tissue can be further classified by location; subcutaneous adipose tissue (SAT) lies beneath the skin, whereas visceral adipose tissue (VAT) lines internal organs, particularly within the mesentery and omentum [59]. They differ profoundly in their metabolic activity, where SAT drains into the systemic circulation, and VAT drains into the portal vein. This difference makes VAT more metabolically active, allowing metabolites and inflammatory factors to reach the liver. Moreover, increased lipolytic activity, in visceral fat, releases free fatty acids that alter hepatic glucose and lipid metabolism. To assess preferential visceral fat storage in an individual, the VAT/SAT ratio can be calculated [60].

Additionally, inflammatory responses in adipose tissue further contribute to peripheral tissue insulin resistance. This occurs through activating stress-induced inflammatory signaling pathways, including JNK, IKKβ, and NF-κB, which promote macrophage infiltration, leading to cytokine secretion that alters insulin signaling [61,62]. Furthermore, dysfunctional adipose tissue secretes adipokines that counteract anti-inflammatory mediators, thereby contributing to ongoing inflammation and metabolic dysregulation [61]. Adipose tissue’s endocrine activity also enables it to affect blood pressure regulation by secreting vasoactivators, leading to endothelial dysfunction, vascular smooth muscle cell proliferation and migration, and vascular inflammation. Adipocytes can additionally produce angiotensinogen, promoting vasoconstriction and fluid retention. Resulting increased circulating free fatty acids and leptin stimulate sympathetic activity and oxidative stress, contributing to hypertension. These factors promote endothelial dysfunction, vascular inflammation, and vascular remodeling, ultimately increasing arterial pressure [63].

### 3.3. Obesity-Induced Glucocorticoid Excess and Metabolic Stress

Obesity, resulting from excess fat accumulation in adipose tissue, specifically VAT, can lead to very serious health problems. It stimulates intracellular pathways that activate multiple biochemical mechanisms in metabolic tissue, including, but not limited to, superoxide generation, phosphorylation reactions, and activation of kinases, all of which contribute to obesity-induced oxidative stress [64,65]. Oxidative stress manifests as a mismatch between the levels of reactive oxygen species and the activity of antioxidant mechanisms, leading to redox signaling dysregulation and molecular damage [66]. Moreover, oxidative stress alters metabolic processes and affects key metabolic tissues, such as the liver, adipose tissue, and endocrine organs, by damaging pancreatic β-cells and interfering with insulin signaling pathways in peripheral tissues, including skeletal muscle. The combination of insulin receptor signaling inhibition and β-cell dysfunction leads to the development of T2DM [65].

Furthermore, metabolic stress induces altered steroidogenesis. This is due to the enhanced activity of the enzyme 11β-hydroxysteroid dehydrogenase (11β-HSD), which is elevated in adipose tissue and, thus, in obese individuals. This enzyme amplifies glucocorticoid action in the liver, muscle, and adipose tissue, even when circulating cortisol levels are normal [67]. Additionally, as cortisol activates both mineralocorticoid and GRs, it enhances mineralocorticoid signaling, which increases aldosterone and RAAS activity, promoting sodium retention, vascular dysfunction, and the development of hypertension [67].

## 4. Steroidogenic Enzyme CYP17A1 Influences Diabetes and Metabolic Disorders

### 4.1. An Overview of Steroidogenesis

Steroidogenesis begins with cellular acquisition of cholesterol, primarily through receptor-mediated uptake of circulating low-density lipoproteins (LDL), although a portion can also be synthesized de novo from acetate within steroidogenic tissues [68,69] StarD4, a ubiquitously expressed sterol-regulated StAR domain protein [70], mediates intracellular cholesterol trafficking to the inner mitochondrial membrane [70,71,72]. CYP11A1 then catalyzes the conversion of cholesterol to pregnenolone, the common precursor of all steroid hormones [73]. Following pregnenolone synthesis, steroidogenesis proceeds through a series of enzymatic reactions within the smooth endoplasmic reticulum and mitochondria, where CYP17A1 expression levels, amongst other enzymes, play a role in final steroid production.

### 4.2. The Role of CYP17A1 in Metabolic Disorders

Metabolic and endocrine stressors like obesity, insulin resistance, and chronic inflammation are known to alter steroidogenic pathways. As CYP17A1 plays a pivotal role in glucocorticoid and androgen biosynthesis, disruptions in endocrine signaling pathways may lead to downstream effects on steroidogenesis, which in turn manifest as systemic metabolic dysfunction. Many studies have identified correlations between variations or disruptions in the CYP17A1 pathway and metabolic dysfunction, as summarized in Table 1.

### 4.3. A Summary of the Function of CYP17A1 in Metabolic Disorders

Collectively, these studies demonstrate that CYP17A1 genetic variation is consistently linked to both metabolic and vascular phenotypes. Beyond its established role in steroidogenesis, CYP17A1 polymorphisms are associated with increased arterial stiffness in diabetes and with susceptibility to T2DM. Together, these findings could support a broader role for CYP17A1 in cardiometabolic disease beyond endocrine steroid production; it is evident that CYP17A1 modulation occupies important functions at the intersection of GC biosynthesis, androgen overproduction, hypertension, metabolic syndrome, and T2DM.

## 5. CYP17A1 Regulates Diabetes Phenotypes

### 5.1. Genetic Variants of CYP17A1 Polymorphisms and Type 2 Diabetes Susceptibility

Given the central role of CYP17A1 in adrenal steroidogenesis and its established contribution to blood pressure regulation and CVD risk, it is biologically plausible that genetic variation at this locus may extend to broader metabolic risk. As previously stated, GCs are critical regulators of hepatic gluconeogenesis, insulin sensitivity, adipocyte differentiation, and pancreatic β-cell function. Even modest alterations in steroidogenic flux may therefore influence glucose homeostasis. For instance, in a case–control study of 440 individuals with T2DM and 1320 controls from a Han Chinese population, Wang et al. investigated three CYP17A1 polymorphisms (rs1004467, rs17115149, and rs12413409). Stratified analyses revealed that the CYP17A1 rs1004467 polymorphism was associated with increased arterial stiffness in diabetic patients, measured by augmentation index [84]. Polymorphisms within the CYP17A1 locus were significantly associated with T2DM risk and glycemic traits. rs17115149 (C > T) conferred increased risk of diabetes in patients with hypertension, and rs12413409 (G > A) was correlated with higher plasma glucose levels in untreated participants, particularly in men. Furthermore, increased CYP17A1 gene expression was observed in T2DM patients, supporting a functional relationship between CYP17A1 activity and dysglycemia [79].

Although currently limited to a single population-based study, these findings suggest that CYP17A1 polymorphisms may contribute to glucose dysregulation in a context-dependent manner, potentially through altered glucocorticoid biosynthesis or tissue-specific steroid metabolism. Importantly, the overlap between hypertension-and diabetes-associated variants at this locus raises the possibility that CYP17A1 represents a shared genetic node linking cardiometabolic traits. Another study revealed that carriers of specific CYP17A1 polymorphisms (e.g., rs6163) show significantly different rates of steroidogenic output [75]. This suggests that individual variations in CYP17A1 activity function as a primary determinant of hormonal flux, potentially amplifying downstream metabolic consequences.

Further replication and mechanistic studies are warranted to clarify whether CYP17A1 variation exerts pleiotropic effects across the spectrum of metabolic disease. Recent findings have also associated PKC-MAPK signaling with cardiac sympathetic complications in T2DM [85], necessitating further studies to highlight the extent of CYP17A1′s role in cardiovascular complications. Moreover, given that current associations are derived largely from observational genetic studies in specific populations (e.g., Han Chinese), with some signals detected only in men, replication across diverse ethnic populations, functional validation of the implicated variants, and explicit consideration of sex-specific effects are needed before these polymorphisms can be proposed as candidates for clinical or precision-medicine application.

### 5.2. Hepatic CYP17A1 Dysfunction Relates to Diabetic Phenotypes

The influence of CYP17A1 on DM is tissue-specific and complex. Physiological context suggests that CYP17A1 may play a dual role in hepatic metabolism. Under fasting conditions, mild increases in hepatic CYP17A1 expression align with the normal rise in gluconeogenic gene expression needed to maintain glucose availability [86]. However, in hepatic insulin resistance (HIR), where insulin fails to suppress gluconeogenic flux while lipogenic pathways remain relatively preserved or even enhanced, emerging evidence implicates ectopic steroidogenic signaling, specifically via CYP17A1, as a mechanistic contributor to selective HIR [30]. Additionally, recent work has revealed that hepatic CYP17A1 becomes aberrantly upregulated in obesity [30,86]. In both diet-induced and genetic rodent models of obesity, hepatic Cyp17a1 expression increases markedly, a response that parallels elevations in its metabolic product 17-OHP in liver tissue and circulation. Similar elevations in hepatic CYP17A1 transcripts have been observed in humans with obesity, with expression levels tracking positively with markers of impaired glycemic control [78,87]. However, it should be noted that because this obesity-associated upregulation of hepatic CYP17A1 is drawn primarily from diet-induced and genetic rodent models; because rodent adrenal and hepatic steroidogenesis differ from the human pathway in several respects (e.g., corticosterone rather than cortisol as the principal glucocorticoid), direct extrapolation of this mechanism to human hepatic physiology should be made cautiously, pending further validation in human tissue. It is also worth noting that cortisol and corticosterone, while structurally related, are not functionally interchangeable glucocorticoids (they differ in receptor affinity and metabolic potency), which further limits how directly rodent hepatic findings can be read onto human physiology.

Nevertheless, findings suggest a conserved hepatic response in metabolic stress whereby CYP17A1 becomes pathologically activated [78,79]. Inhibition of CYP17A1 activity has also been found to profoundly ameliorate metabolic dysfunction. RNA interference–mediated depletion of hepatic Cyp17a1 in obese diabetic mice lowers circulating glucose and insulin levels, improves glucose and insulin tolerance, enhances insulin signaling, reduces hepatic lipid accumulation, and lowers liver injury markers [30]. Thus, hepatic CYP17A1 functions as a context-dependent metabolic node: Adaptive under fasting conditions yet potentially maladaptive when chronically activated in obesity. This duality reinforces the importance of temporal and quantitative regulation in determining whether CYP17A1 activity supports metabolic flexibility or drives pathological HIR.

A mechanistic link between heightened CYP17A1 expression and glucose dysregulation appears to be mediated by the unexpected ability of 17-OHP to function as an endogenous ligand for the GR. Augmenting the hepatic CYP17A1/17-OHP axis exacerbates metabolic dysfunction. Overexpression of CYP17A1 in the liver elevates blood glucose levels, worsens glucose tolerance, diminishes insulin sensitivity, and amplifies hepatic gluconeogenesis. Mice receiving exogenous 17-OHP develop similar disturbances despite unchanged body weight or food intake, highlighting that the metabolic impairments arise directly from altered endocrine metabolic signaling rather than changes in energy balance. These effects coincide with increased hepatic TG accumulation and suppressed insulin signaling, demonstrating that the CYP17A1 pathway contributes to both glucose overproduction and lipid dysregulation [30].

Importantly, the metabolic disturbances induced by 17-OHP are abolished when hepatic GR is knocked down, establishing GR as the critical effector through which CYP17A1 exerts its influence on glucose homeostasis [30]. This establishes a novel local CYP17A1–17-OHP–GR axis in the liver that integrates metabolic and endocrine signals, highlighting how tissue-specific steroid metabolism and receptor-level modulation can drive features of metabolic syndrome. By linking altered adrenal precursor flux to intrahepatic GR activation, this framework positions CYP17A1 as a key mechanistic node connecting nutrient excess, steroid intermediates, and metabolic dysfunction [30].

Collectively, current evidence supports a model in which obesity-associated upregulation of hepatic CYP17A1 sustains insulin-independent gluconeogenesis. This steroid-driven transcriptional dominance explains persistent hepatic glucose production, despite hyperinsulinemia, and provides a molecular framework for selective HIR. Secondary consequences, including hepatic steatosis, lipotoxic signaling, and amplification of inflammatory pathways, may further entrench systemic insulin resistance and accelerate progression toward T2DM. Rather than directly impairing insulin receptor signaling cascades, CYP17A1 reshapes the intrahepatic steroid milieu, integrating endocrine and metabolic signals into a transcriptional program that favors glucose overproduction. This conceptual distinction is critical: CYP17A1 acts as an upstream steroidogenic amplifier of counter-regulatory signaling rather than a proximal inhibitor of insulin action.

### 5.3. Peripheral Insulin Resistance and CYP17A1-Mediated Endocrine–Metabolic Crosstalk

Perturbations in steroidogenic enzyme activity can remodel the metabolic phenotype independently of canonical insulin signaling, highlighting steroid biosynthesis as a potential driver of peripheral insulin resistance and metabolic disease susceptibility [88]. Dysregulation of CYP17A1 activity is poised to influence peripheral insulin sensitivity, positioning the enzyme as a compelling target for mechanistic investigation and potential therapeutic intervention [79].

Emerging evidence has shown that CYP17A1 deficiency may confer transient metabolic protection. In a CRISPR-Cas9 Cyp17a1 knockout rat model assessed over 20 weeks, Cyp17a1 loss preserved systemic insulin sensitivity despite the development of obesity [18]. Insulin tolerance tests demonstrated intact insulin signaling, circulating insulin remained physiological, and glucose disposal was uncompromised. Mechanistically, this protection was attributed to adipose tissue remodeling, favoring subcutaneous fat accumulation over visceral depots, where knockout animals exhibited a significantly elevated SAT:VAT ratio [89]. Given the strong association between visceral adiposity, systemic inflammation, ectopic lipid flux, and insulin resistance, this redistribution likely underpins preserved insulin sensitivity [90].

Prolonged CYP17A1 deficiency, however, precipitates progressive metabolic dysfunction. In Cyp17a1^−/−^ mice monitored to 12 months, a divergent trajectory emerged, characterized by central obesity, increased visceral fat, hepatic steatosis, and features consistent with metabolic syndrome. VAT expansion and inflammation, as evidenced by crown-like structures, coincided with dysregulated steroidogenesis, including elevated aldosterone and corticosterone, diminished sex steroids (DHEA, DHEAS, estradiol), hyperinsulinemia, and elevated resistin. These changes were accompanied by hyperglycemia, impaired glucose tolerance, and blunted insulin responses, confirming systemic insulin resistance. Systolic blood pressure elevation further reinforced the metabolic syndrome phenotype [78].

Whilst the metabolic consequences reported across the two models differ markedly, the ~20-week-old Cyp17a1 knockout rats and the ~12-month-old Cyp17a1^−/−^ mice represent congenital, lifelong CYP17A1 deficiency rather than a true short-term versus chronic intervention. Therefore, these findings should not be interpreted as direct longitudinal evidence of a time-dependent progression within a single model. The divergent phenotypes more likely reflect differences in species, genetic background, age, and sex between the two cohorts, among which a shift toward mineralocorticoid overproduction in the older cohort is one plausible, though as of yet unconfirmed, contributor. It is also worth noting that, because both are congenital rather than inducible knockouts, chronic compensatory adjustments in upstream regulators (e.g., ACTH, the renin–angiotensin system) may themselves shape the observed steroid and metabolic phenotype over the animal’s lifetime, independent of any acute, direct consequence of CYP17A1 loss—a caveat inherent to constitutive knockout models generally. In any case, excess aldosterone activates mineralocorticoid receptors (MR) in insulin-sensitive tissues, inducing oxidative stress, inflammation, and impaired insulin signaling—hallmarks of peripheral insulin resistance [91,92]. Notably, conditions of CYP17A1 hyperactivity, such as PMOS, demonstrate severe insulin resistance in skeletal muscle and adipose tissue, with 17-OHP levels correlating directly with insulin resistance severity independent of BMI [93,94,95,96]. This evidence supports a model in which CYP17A1-derived steroid intermediates directly impair peripheral insulin sensitivity.

Emerging data indicate that CYP17A1 activity is modulated by insulin signaling, creating the potential for pathological feed-forward amplification. Hyperinsulinemic–euglycemic clamp studies in hyperandrogenic women show that elevated insulin enhances ACTH-driven adrenal steroidogenesis, increasing 17-hydroxylated intermediates while selectively modulating CYP17A1 catalytic balance [97]. Hyperinsulinemia preferentially amplifies 17α-hydroxylase activity while attenuating 17,20-lyase function, effectively reshaping steroid flux *in vivo*. This establishes a vicious cycle: peripheral insulin resistance → compensatory hyperinsulinemia → augmented CYP17A1 activity → increased production of insulin resistance-promoting steroids → exacerbated peripheral insulin resistance. Such a feed-forward loop may underlie the progressive insulin resistance observed in steroidogenic disorders, suggesting that pharmacological targeting of CYP17A1 could disrupt this deleterious amplification.

### 5.4. CYP17A1-Mediated Steroid Signaling in Adipose Tissue Dysfunction and Diabetes

Adipose tissue represents a more immediate mechanistic interface through which alterations in CYP17A1-derived steroid intermediates can influence metabolic phenotype [18,77,78]. As a steroid-sensitive and metabolically adaptive organ capable of peripheral steroid modification, adipose depots respond robustly to shifts in androgenic and glucocorticoid tone that govern adipocyte differentiation, lipid partitioning, inflammatory signaling, and insulin responsiveness [98,99,100,101,102,103]. Consequently, perturbations in CYP17A1 activity are more readily expressed through adipose remodeling and dysfunction than through skeletal muscle signaling cascades. These positions adipose tissue as a logical focal substrate for examining steroid-mediated contributions to peripheral insulin resistance.

Rodent studies have demonstrated expression of Cyp17a1, alongside the full complement of steroidogenic enzymes in both visceral and subcutaneous depots, with detectable intratissue testosterone production and conversion of pregnenolone to androgens by preadipocytes and mature adipocytes. Importantly, obesity was associated with coordinated upregulation of these enzymes, indicating that adipose tissue contributes to systemic steroid milieu and provides a substrate through which CYP17A1-dependent signaling may influence adipose remodeling and insulin sensitivity [104].

Jeon et al. moreover reported that in young Cyp17a1-knockout rats (≈20 weeks), fat mass increases were largely confined to subcutaneous depots and insulin sensitivity remained intact [18]. By contrast, studies of aged knockout mice (≈12 months) found full metabolic-syndrome phenotypes—central obesity, visceral adipose expansion, fatty liver, hyperinsulinemia and insulin resistance—accompanied by glucocorticoid excess and depletion of sex steroids [78]. These apparently paradoxical outcomes may potentially be explained by a time-dependent shift in adipose tissue [105,106,107,108]; however, as noted above, this interpretation is drawn from two distinct congenital knockout models differing in species and age rather than from a single longitudinal cohort, and should be regarded as a hypothesis rather than established evidence. One possibility is that acute Cyp17a1 loss favors adipose redistribution toward ‘healthy’ subcutaneous depots (preserving insulin action), whereas prolonged congenital deficiency is associated with pathological VAT expansion, inflammation, and endocrine imbalance that drive insulin resistance over time. The evolving steroidogenic profile of CYP17A1-deficient animals (e.g., rising aldosterone/corticosterone with low androgens/estrogens) likely underlies this progression, tipping adipose tissue from an initially benign state into dysfunction.

Furthermore, the reach of the CYP17A1–17-OHP–GR axis extends beyond the liver. The dysregulation of CYP17A1 also fundamentally alters adipose tissue architecture and function, further cementing the state of metabolic syndrome. Like the liver, adipose tissue expresses the GR, making it highly sensitive to the local steroid environment. In the presence of nutrient excess, if CYP17A1 produces 17-OHP locally within fat cells, it can activate the GR and mimic the effects of chronic cortisol exposure—a phenomenon often described as “Cushingoid” fat distribution [104,109,110,111]. The validity of this “local amplification” model is supported by studies of 11β-HSD1. In mice lacking adipose 11β-HSD1, visceral fat accumulation is reduced, and insulin sensitivity is improved [112].

Similarly, human clinical data show that inhibiting local cortisol generation in adipose tissue directly decreases glucocorticoid-driven lipolysis [113]. Within the context of CYP17A1, these findings suggest that adrenal precursor flux, when coupled with tissue-specific regeneration via 11β-HSD1 or intermediate accumulation (like 17-OHP), can potentiate adipose dysfunction [114]. By linking altered adrenal precursor flux and hepatic gluconeogenesis to this destructive remodeling of adipose tissue, the CYP17A1–17-OHP axis emerges as a unified mechanistic node. It connects nutrient excess to a multi-organ failure of steroid and metabolic homeostasis, where the liver drives hyperglycemia and adipose tissue drives systemic inflammation and central obesity [30,115,116]. Overall, direct evidence that CYP17A1-derived steroid intermediates drive adipose dysfunction specifically in humans remains limited: the mechanistic model outlined above is derived largely from rodent and *in vitro* work, and the extent to which hepatic versus adipose contributions, and animal versus human evidence, can be cleanly separated within this axis, remains to be established.

## 6. CYP17A1 Is Involved in Different Metabolic Pathways

### 6.1. Classical Steroidogenic Pathway and Δ5 and Δ4 Pathways

**Figure 2:**
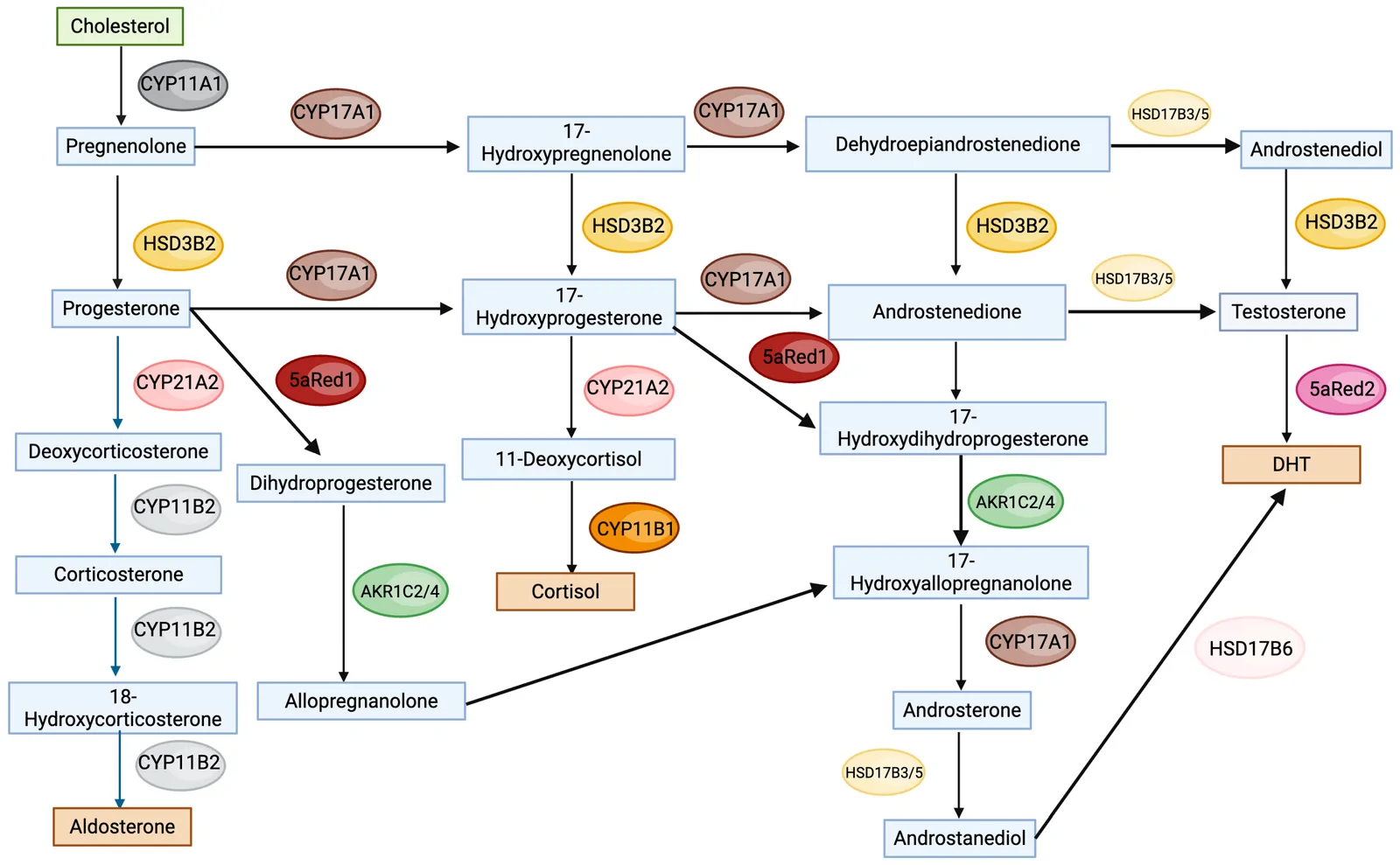
Steroidogenesis pathway, including the backdoor pathway for sex steroid production. 17-OHP: 17-Hydroxyprogesterone; 17-OHPreg: 17-Hydroxypregnenolone; 5αRed1: 5-Alpha-Reductase Type 1; 5αRed2: 5-Alpha-Reductase Type 2; AKR1C2/4: Aldo-Keto Reductase Family 1 Member C2 and Member C4; CYP11A1: Cytochrome P450 Family 11 Subfamily A Member 1; CYP11B2: Cytochrome P450 Family 11 Subfamily B Member 2; CYP17 A1: Cytochrome P450 Family 17 Subfamily A Member 1; CYP21A2: Cytochrome P450 Family 21 Subfamily A Member 2; DHEA: Dehydroepiandrosterone; DHT: Dihydrotestosterone; HSD17B3/5: 17β-Hydroxysteroid Dehydrogenase Type 3 and Type 5; HSD17B6: 17β-Hydroxysteroid Dehydrogenase Type 6: HSD3B2: 3β-Hydroxysteroid Dehydrogenase Type 2. Created in BioRender. Nandakumar, M. (2026), https://BioRender.com/ibt74rc.

The steroidogenesis pathway is a complex, interconnected metabolic network carried out in various sites, including the adrenal gland and gonads, amongst other tissues. Its regulation is controlled by factors such as precursor availability and tissue-specific enzyme expression. The main products of this pathway include mineralocorticoids, such as aldosterone, glucocorticoids, such as cortisol, and sex steroids, including testosterone and estradiol. Steroid hormones play central roles in multiple bodily functions, including regulating glucose metabolism and insulin sensitivity, where some metabolic disturbances have been related to changes in cortisol levels [117]. CYP17A1, along with other enzymes, is important in the regulation and production of steroids through this pathway [32]. A summary of the steroidogenesis pathway can be found in Figure 2.

Steroidogenesis begins following the translocation of cholesterol from the cytoplasm into the mitochondria by steroidogenic acute regulatory protein (StAR) [118], which is converted into pregnenolone by CYP11A1 [33]. Then, pregnenolone is converted to either progesterone (Δ4 pathway) or 17-hydroxypregnenolone (17-OHPreg) (Δ5 pathway). The direction of the pathway is dependent on the site-specific differential enzyme expression within the different layers of the adrenal tissue: zona glomerulosa, zona fasciculata, and zona reticularis. The enzymatic composition in various tissues determines the direction of steroidogenesis [34].

#### 6.1.1. CYP17A1 in Mineralocorticoid Synthesis

The primary site for mineralocorticoid production upon signal activation by angiotensin II is in the zona glomerulosa [34]. In this layer, there is a lack of CYP17A1 expression and an abundance of HSD3B2, which leads to the conversion of pregnenolone to progesterone instead of the alternative 17-OHPreg [32]. Due to the lack of CYP17A1, progesterone then undergoes multiple conversions by other enzymes, where it is ultimately transformed to aldosterone.

#### 6.1.2. CYP17A1 in Glucocorticoid Synthesis

The main site for glucocorticoid production upon signal activation by the trophic protein hormone ACTH is in the zona fasciculata in the adrenal cortex [34], where there is an abundance of CYP17A1 and HSD3B2. ACTH stimulates CYP17A1 expression in the zona fasciculata and zona reticularis via the melanocortin 2 receptor (MC2R) receptor, thereby activating the cAMP/PKA signaling cascade. As a result, there is an enhancement of transcription of steroidogenic enzymes involved in glucocorticoid biosynthesis [35,36,37].

**Figure 3:**
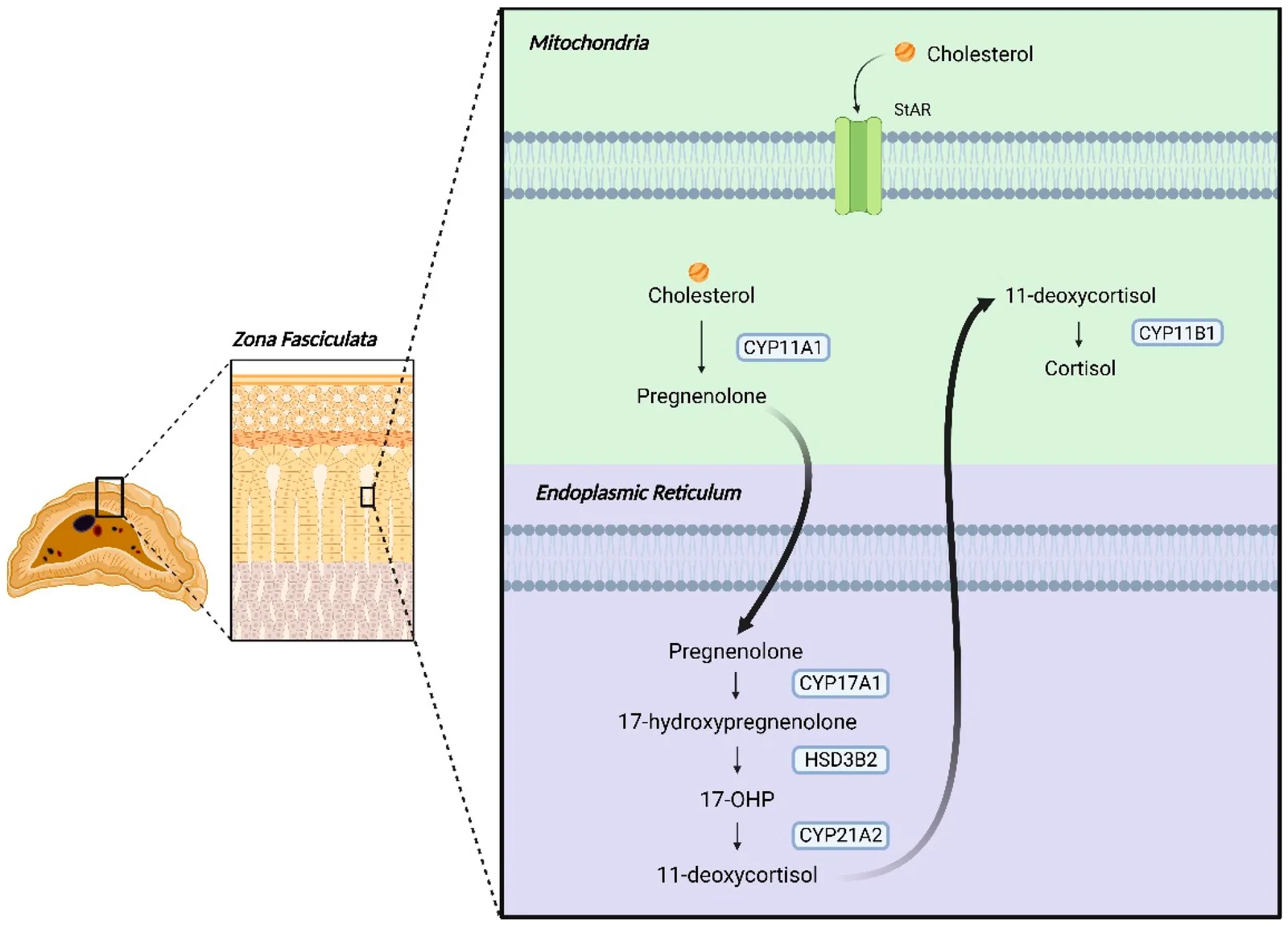
Compartmentalized steroidogenic pathway leading to cortisol synthesis in adrenal zona fasciculata cells. Overview of steroidogenic conversion of cholesterol to cortisol, illustrating the subcellular localization of enzymatic steps within mitochondria and smooth endoplasmic reticulum and the tissue context of the adrenal cortex zona fasciculata. Cholesterol transport into mitochondria is mediated by steroidogenic acute regulatory protein (StAR), followed by mitochondrial side-chain cleavage to pregnenolone. Pregnenolone translocates to the endoplasmic reticulum for sequential hydroxylation and dehydrogenation reactions producing 17-hydroxypregnenolone, 17-hydroxyprogesterone, and 11-deoxycortisol, which re-enters mitochondria for terminal hydroxylation to cortisol. Arrows denote metabolite trafficking between organelles and enzyme labels indicate catalytic steps. StAR: Steroidogenic Acute Regulatory Protein; CYP11A1: Cholesterol Side-Chain Cleavage Enzyme; CYP17A1: 17α-Hydroxylase/17,20-Lyase; HSD3B2: 3β-Hydroxysteroid Dehydrogenase Type 2; CYP21A2: 21-Hydroxylase; CYP11B1: 11β-Hydroxylase; 17-OHP: 17-Hydroxyprogesterone. Created in BioRender. Nandakumar, M. (2026), https://BioRender.com/acbonbi.

Due to the competing presence of CYP17A1, pregnenolone does not result in mineralocorticoid production. Instead, it is converted to 17-OHPreg by CYP17A1 17α-hydroxylation and is then converted to 17-hydroxyprogesterone (17-OHP) by HSD3B2. Alternatively, pregnenolone can also be converted directly to progesterone by HSD3B2, which is then hydroxylated to 17-OHP by CYP17A1 [119]. 17-OHP is then converted to 11-deoxycortisol by CYP21A2, which is finally converted to cortisol by CYP11B1, as shown in Figure 3 [120]. 17,20-lyase activity is limited in the zona fasciculata due to the scarcity of CYB5, which is needed for efficient electron transfer to cleave carbon-carbon bonds [20,21,121,122].

#### 6.1.3. CYP17A1 in Sex Steroid Synthesis

In the production of sex steroids, LH, through a similar cAMP/PKA signaling cascade, activates sex steroidogenesis in the zona reticularis and in gonads [35], where there is low expression of HSD3B2 and large quantities of CYB5. Therefore, CYP17A1 17,20 lyase converts 17-OHPreg to DHEA [120]. However, there are still minimal traces of 17-OHP conversion by HSD3B2, which CYP17A1 then converts to androstenedione by its 17,20 lyase activity. Both byproducts are then converted to testosterone in Leydig cells and finally to dihydrotestosterone (DHT), the biologically active form of testosterone [119]. In female reproductive cells, testosterone is converted to estradiol [123].

Alternatively, in the testes, there have been studies that found a backdoor pathway to DHT production that does not require DHEA or testosterone. The backdoor pathway also has two sub-pathways depending on its continuation on the Δ4 pathway or Δ5 pathway. If undertaking the Δ4 pathway, pregnenolone is converted to progesterone; however, 5aRed1 then converts it to dihydroprogesterone (DHP). Following multiple conversions by AKR1C2/4, CYP17A1 hydroxylase, CYP17A1 17,20 lyase, and HSD17B3/5 enzymes, DHP is converted to allopregnenolone, 17-OH-allopregnenolone, androsterone, and androstanediol, respectively. Androstanediol is then converted to DHT by HSD17B6. DHP can also be converted to 17-OH-dihydroprogesterone by CYP17A1, then AKR1C2/4 which converts it to 17-OH-allopregnenolone. Also following in the Δ4 pathway, CYP17A1 can act on progesterone to produce 17-OHP, which is then converted to 17-OH-dihydroprogesterone by 5aRed1, where it then continues to DHT as mentioned previously. In the Δ5 pathways, where pregnenolone is converted to 17-OHPreg by CYP17A1 first, HSD3B2 converts it to 17-OHP, and due to the presence and activity of 5aRed1, 17-OHP is then converted to 17-OH-dihydroprogesterone and continues along the previous path to DHT [124].

### 6.2. cAMP/PKA and PI3K/Akt in the Expression of CYP17A1

Due to the relevance of CYP17A1 expression in the steroidogenic pathway, it is important to investigate the activation signal pathways that may contribute to changes in the expression levels of the enzyme in various tissues, such as the gonads and the adrenal cortex. One pathway is the cAMP/PKA pathway, which can be activated by LH or ACTHin their respective tissues. LH binds to LHCGR and activates the cAMP/PKA pathway, resulting in StAR transcription, which is the main protein required to transport cholesterol to the inner mitochondrial membrane, where it is then used in the first step of steroidogenesis [125,126]. Also, ACTH stimulates the cAMP/PKA pathway when bound to MC2R and regulates steroidogenic gene expression, including CYP17A1 [127]. Prostate cancer studies have also demonstrated that CYP17A1 is potentially upregulated by cAMP/PKA signalling through mechanisms involving transcription factor activation such as SF-1 [128].

**Figure 4:**
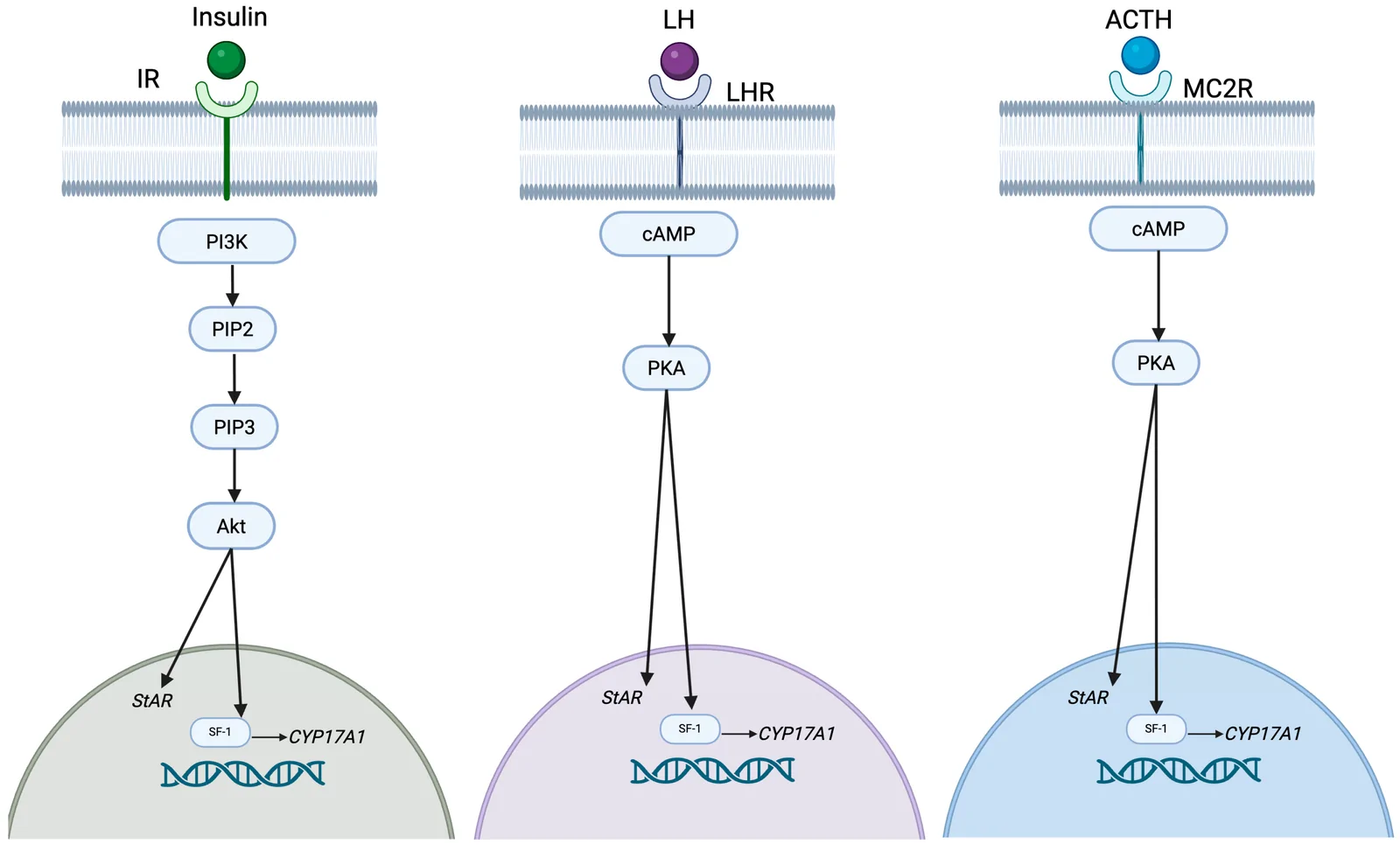
Potential CYP17A1 activation through PI3K/AKT and cAMP/PKA pathways when triggered by tissue-specific stimuli. ACTH: Adrenocorticotropic hormone; Akt: Protein Kinase B; cAMP: cyclic adenosine monophosphate; CYP17A1: Cytochrome P450 Family 17 Subfamily A Member 1; IR: Insulin receptor; LH: Luteinizing hormone; LHR: Luteinizing hormone receptor; MC2R: melanocortin 2 receptor; PI3K: Phosphatidylinositol 3-kinase; PIP2: Phosphatidylinositol 4,5-bisphosphate; PIP3: Phosphatidylinositol (3,4,5)-trisphosphate; PKA: Protein kinase A; SF-1: Steroidogenic factor-1; StAR: Steroidogenic Acute Regulatory protein. Created in BioRender. Nandakumar, M. (2026), https://BioRender.com/ibt74rc.

A secondary pathway in potentially regulating CYP17A1 expression is the phosphatidylinositol 3-kinase/protein kinase B (PI3K/Akt) pathway. Insulin binding to the insulin receptor (IR) activates downstream signaling through insulin receptor substrate (IRS) proteins, leading to PI3K activation. PI3K catalyzes the phosphorylation of PIP2 to PIP3 and, in turn, facilitates Akt activation [129]. Following activation, Akt promotes StAR [130] transcription and phosphorylates SF-1 [89,130] which may implicate this pathway in CYP17A1 activation and expression. The potential CYP17A1 activation pathways are shown in Figure 4. Further investigation is required to establish a complete mechanism for CYP17A1 transcription and regulation by various stimuli in different cell types.

### 6.3. The Hepatic CYP17A1 Fasting Axis

In the physiological feeding state, when there is an increase in glucose circulation in the body, insulin is secreted from pancreatic beta-cells. Glucose uptake in the liver is primarily mediated by GLUT2. Glucose is then metabolized for use or, when in excess, is converted to glycogen for storage [131]. Insulin also promotes glycogen synthesis in the liver and skeletal muscle, facilitating the storage of glucose for future energy requirements [132]. In addition, insulin activates the Akt signaling pathway, through which Akt phosphorylates FOXO1, inhibiting its transcriptional activity. This suppresses the expression of key gluconeogenic genes and contributes to a reduction in hepatic glucose production [133]. In the fasting state, as glucose and insulin levels decline, glucagon secretion increases, activating hepatic glycogenolysis and gluconeogenesis to sustain glucose availability [134]. Additionally, reduced insulin signaling through the PI3K/Akt pathway decreases FOXO1 phosphorylation, allowing FOXO1 to promote the transcription of gluconeogenic genes.

**Figure 5:**
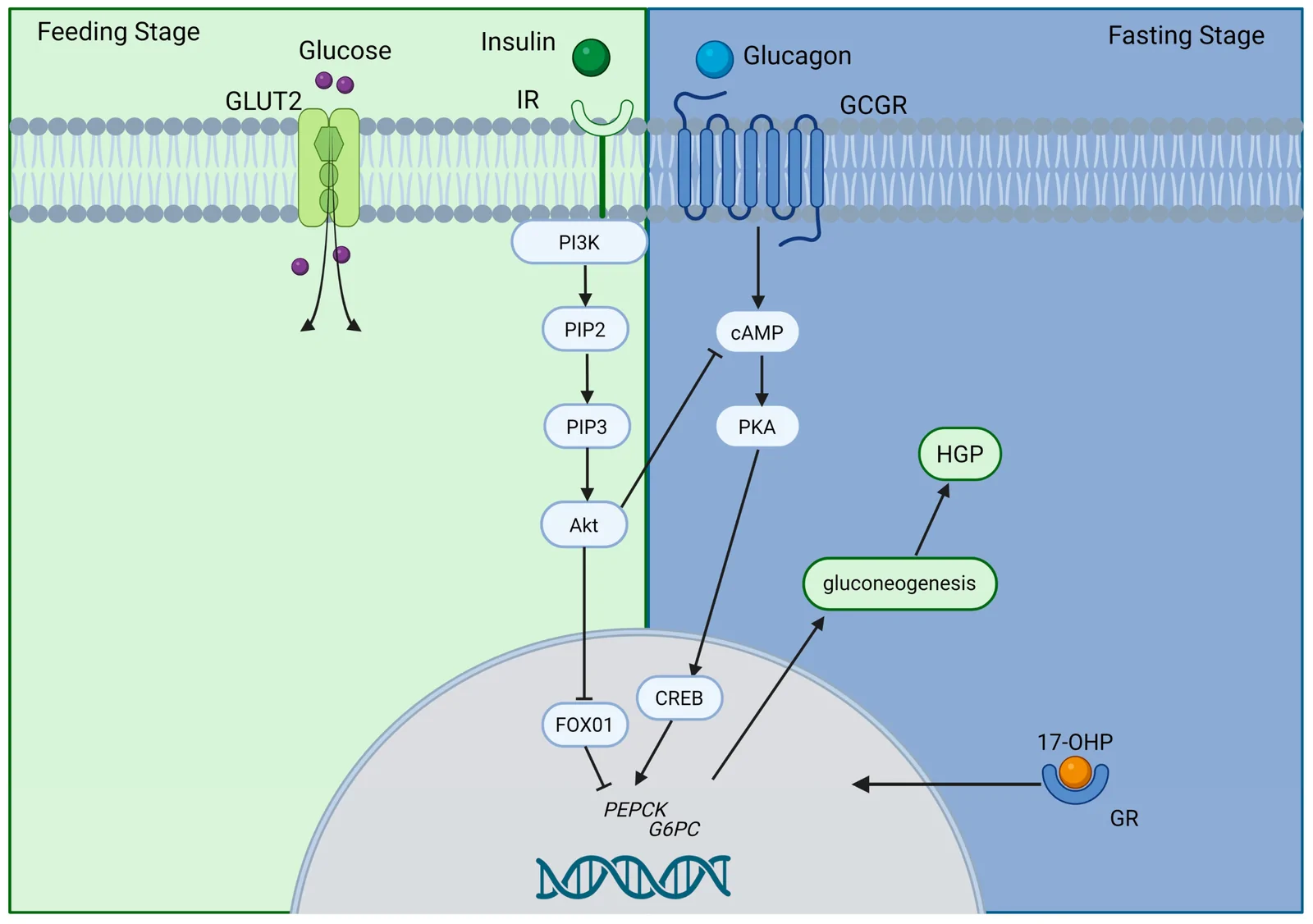
Hepatic fasting and feeding stages and the possible link of 17-OHP in hepatic glucose production. Akt: Protein Kinase B; cAMP: Cyclic adenosine monophosphate; CREB: cAMP response element-binding protein; FOXO1: Forkhead box protein O1; GCGR: Glucagon receptor; GLUT2: Glucose Transporter Type 2; GR: Glucocorticoid receptor; HGP: Hepatic glucose production; G6PC: Glucose-6-phosphatase catalytic subunit 1; IR: Insulin receptor; PI3K: Phosphatidylinositol 3-kinase; PIP2: Phosphatidylinositol 4,5-bisphosphate; PIP3: Phosphatidylinositol (3,4,5)-trisphosphate; PEPCK: Phosphoenolpyruvate carboxykinase; PKA: Protein kinase A; 17-OHP: 17-hydroxyprogesterone. Created in BioRender. Nandakumar, M. (2026), https://BioRender.com/ibt74rc.

CYP17A1 could also play a role in the regulation of gluconeogenesis. An increase in CYP17A1 was found to increase 17-OHP, which then activated GRs [30]. An overview of the hepatic fasting and feeding processes and 17-OHP involvement is presented in Figure 5.

## 7. CYP17A1 and the Efficacy of Diabetes/Metabolic Treatment

### 7.1. The Role of CYP17A1 Inhibitors in Targeting Metabolic Disease

Given CYP17A1′s implication in many pathologies including T2DM, PMOS, prostate cancer, and hypertension, it has emerged as a promising therapeutic target across endocrine and metabolic disease [74,75,76]. As of 2026, the steroid ligand abiraterone remains the only CYP17A1 inhibitor approved for clinical use [135]. The inhibitory mechanism of abiraterone is closely related to the structural features of CYP17A1. Owing to its steroid-like core scaffold, abiraterone can occupy the enzyme’s steroid-binding pocket [136]. At the C17 position, the molecule contains a 3-pyridyl group whose sp^2^-hybridized nitrogen atom coordinates directly with the heme iron, thereby blocking oxygen activation and preventing catalytic activity.

This inhibitor has been pivotal in identifying the regulatory importance of CYP17A1 in governing steroid pathway dynamics, where clinical studies conducted on cohorts with prostate cancer reaffirm that CYP17A1 inhibition with abiraterone results in significant suppression of androgen and cortisol synthesis [137]. However, findings detailing a mechanistic overlap between abiraterone and glycemic regulation in the context of T2DM remain limited. One study depicts that abiraterone and prednisone therapy may be associated with hypoglycemia when administered to prostate cancer patients with T2DM simultaneously receiving glucose-lowering agents [138]. Another study suggests a rare but potential correlation with abiraterone and prednisone therapy and the development of de novo T2DM in prostate cancer patients [139]. Therefore, exploring CYP17A1 inhibition in the context of T2DM could yield interesting correlations that warrant further investigation.

Moreover, despite its clinical efficacy, abiraterone produces substantial endocrine disturbances due to non-selective inhibition of both hydroxylase and lyase activities, leading to compensatory adrenocorticotropic hormone elevation and mineralocorticoid excess [140]. By inhibiting 17α-hydroxylase, abiraterone reduces cortisol synthesis, which triggers compensatory ACTH elevation; the resulting upstream steroid excess is diverted toward mineralocorticoid-active precursors, such as aldosterone, producing hypertension, hypokalemia, and fluid retention [141]. Simultaneous inhibition of 17,20-lyase suppresses DHEA/androstenedione formation and thereby lowers testosterone and downstream estrogen [77,142]. This has been linked to the development of metabolic syndrome, obesity and T2DM, resulting from low-grade inflammation, β-cell stress and chronic hyperglycemia [143,144,145]. Moreover, it has been found that in patients with T2DM, abiraterone–prednisone therapy has been associated with deterioration of glycemic control [138]. The use of abiraterone requires exogenous glucocorticoid replacement, which can adversely affect insulin sensitivity and glycemic control [146]. Thus, the use of prednisone underscores the exquisite sensitivity of hepatic glucose homeostasis to steroid balance within the CYP17A1 axis and leads to further metabolic dysregulation.

Whilst monitoring blood pressure, fluid retention, and potassium levels is frequently combined with abiraterone therapy, overall, these treatments fail to address the cause of steroid imbalance caused by this inhibitor, highlighting a key limitation of the drug [140]. Therefore, these limitations served as a motive to dissociate the suppression of androgen synthesis from the impairment of glucocorticoid production. As a result, more selective CYP17A1 inhibitors have been developed, including both steroidal and non-steroidal scaffolds designed to preferentially inhibit 17,20-lyase activity whilst sparing hydroxylase function. These inhibitors include non-steroidal pyridyl alkyl adamantane carboxylates [147], benzothiophene core inhibitors [148], and Seviteronel (VT-464) and Orteronel (TAK-700), which preferentially inhibit the lyase reaction [149,150].

A steroidal alternative to abiraterone is galeterone, which has now cleared phases I and II of clinical trial [151]. It selectively inhibits the 17,20 lyase activity of CYP17A1 [152], resulting in no side effects of mineralocorticoid excess and glucocorticoid deficiency. Though some adverse events observed included nausea, fatigue, and abdominal pain, as well as isolated cases of hypokalemia and peripheral edema, these events were not clustered with other features of mineralocorticoid excess and did not reflect a consistent endocrine toxicity pattern [151]. As a result, galeterone was administered without the need for concomitant glucocorticoid supplementation [153]. Thus, the preservation of 17α-hydroxylase activity in lyase-selective inhibitors has set promising groundwork to produce novel CYP17A1 inhibitors that can also maintain physiologic cortisol homeostasis and prevent HPA-axis activation.

### 7.2. Glucocorticoid-Receptor-Targeted Therapies

As previously discussed, 17-OHP can activate hepatic GR signaling, promoting GR nuclear activity and inducing a GR-dependent transcriptional program that upregulates gluconeogenic genes, thereby increasing hepatic glucose production and worsening insulin resistance [30]. As GR overactivation is a well-established driver of hyperglycemia and impaired glucose tolerance, evidence in T2DM supports impaired glucocorticoid negative feedback (relative GC resistance) with HPA-axis hyperactivity; individuals in higher quartiles of 17-OHP exhibited higher fasting and 2 h glucose, HbA1c, triglycerides, and free fatty acids [30]. Crucially, GR blockade has been shown to functionally reverse this metabolic phenotype: Mifepristone suppressed 17-OHP-driven GR target gene induction and attenuated 17-OHP-induced hyperglycemia and insulin resistance, supporting GR as a proximal effector of 17-OHP metabolic toxicity [30].

While increased hepatic CYP17A1 expression appears to drive pathological 17-OHP accumulation in metabolic disease, as previously stated, systemic CYP17A1 inhibition is associated with significant disruption of cortisol and sex steroid synthesis, including mineralocorticoid excess and hypogonadism. As 17-OHP production arises from the enzyme’s 17α-hydroxylase activity, modulation of this pathway would require careful consideration of endocrine consequences. In parallel, GR antagonism represents a downstream strategy with established clinical precedent: GR blockade has been shown to reverse 17-OHP–induced gluconeogenic gene expression and metabolic dysfunction in preclinical models, and GR antagonists such as mifepristone improve hyperglycemia in hypercortisolemic states. Together, these data suggest that targeting both upstream CYP17A1 activity and downstream GR signaling may provide complementary metabolic benefit by reducing 17-OHP production while simultaneously blocking its hepatic glucocorticoid-like effects. Such a dual approach could theoretically attenuate pathological gluconeogenic signaling whilst minimizing the systemic endocrine disruption observed with broad CYP17A1 inhibition alone.

### 7.3. Precision Medicine Based on CYP17A1 Variants

Across multiple cohorts, genetic variation within the CYP17A1 pathway highlights the complex interface between steroidogenesis and cardiometabolic risk. As mentioned above, polymorphisms rs17115149 (C > T) and rs12413409 (G > A) within the CYP17A1 locus were significantly associated with T2DM risk and glycemic traits, and in diabetic subjects, the CYP17A1 rs1004467 polymorphism was associated with increased arterial stiffness [84]. Notably, the latter association was observed primarily in individuals not receiving antihypertensive therapy, as opposed to those who were receiving antihypertensives, suggesting a genotype-dependent vascular phenotype that may be modified by pharmacologic treatment [84].

Pharmacogenetic evidence further illustrates that CYP17A1 variation influences clinically meaningful steroidogenic outcomes. In patients with Cushing’s syndrome treated with steroidogenesis inhibitors (ketoconazole and/or metyrapone), *rs6163* polymorphism in CYP17A1 significantly modified therapeutic response [75]. Individuals homozygous for the C allele (CC genotype) were nearly four times more likely to achieve normalization of urinary free cortisol compared with AA/AC carriers. Carriers of the CC genotype also required a shorter duration of treatment to reach eucortisolism, suggesting enhanced pharmacologic sensitivity [75]. Given that chronic GC excess is a well-established driver of insulin resistance, central adiposity, and hyperglycemia, targeting genetic variants that modulate steroidogenic flux and cortisol normalization can have important downstream metabolic implications [154,155].

### 7.4. Emerging Small-Molecule CYP17A1 Modulators

Recent advances in virtual screening and structure-guided medicinal chemistry have identified a growing number of plant-derived and synthetic small molecules capable of modulating CYP17A1 activity. Curcuminoids were found to significantly inhibit CYP17A1 activity in human adrenal NCI-H295R cells, reducing both 17α-hydroxylase and 17,20-lyase reactions in a concentration-dependent manner [156]. At 10 µg/mL, curcuminoids suppressed both enzymatic activities to a degree comparable to 1 µM abiraterone under the same experimental conditions [156]. Green tea catechins and black tea theaflavins have also been identified as natural inhibitors of CYP17A1 activity *in vitro*, with gallate-containing polyphenols demonstrating measurable suppression of C17,20-lyase activity [157]. Structure–activity analysis suggested that the presence of a pyrogallol (gallate) moiety was critical for inhibitory potency, as compounds lacking this feature showed minimal effect on enzyme activity [157]. Notably, incorporating these holistic molecules into metabolic disorder treatment can act as a starting point for multiplex approaches to treating T2DM, through targeting CYP17A1 in a way that overcomes current pitfalls.

Recent work has identified dihydrotanshinone I, an abietane diterpenoid quinone derived from Salvia miltiorrhiza, as a selective inhibitor of CYP17A1 17,20-lyase activity *in vitro* [158]. In contrast to abiraterone, which non-selectively suppresses both lyase and 17α-hydroxylase functions and necessitates glucocorticoid co-administration, dihydrotanshinone I largely preserved hydroxylase activity [158]. Recently, in silico analysis has discovered 6-(1,1-dimethylallyl) genistein, 4-hydroxytamoxifen (4-OHT) analog 13, and isowighteone as top candidates for CYP17A1 inhibition, with docking scores comparable to or exceeding the reference inhibitor TOK-001 [159]. Although all three compounds demonstrated favorable docking profiles, only 4-hydroxytamoxifen analog 13 and 6-(1,1-dimethylallyl) genistein maintained consistently strong binding energies and stable molecular dynamics behavior [159]. Nevertheless, while these computational candidates have demonstrated CYP17A1 binding activity, further studies are necessary to establish the inhibitory, selectivity and pharmacokinetics of the compounds, to confirm if they can be safely used as CYP17A1 inhibitors. Only then could they potentially be investigated for therapeutic applications within the context of metabolic disorders.

## 8. Conclusions and Future Perspectives

Based on the evidence presented in this review, it is evident that CYP17A1 has emerged as a potential therapeutic target of metabolic disorders, particularly through its effects on steroid biosynthesis, glucose homeostasis, adipose dysfunction, and blood pressure regulation. Its role in controlling the balance of glucocorticoids, mineralocorticoids, and sex steroids places CYP17A1 at a critical intersection between endocrine signaling and metabolic disease [30,86].

The evidence discussed in this review suggests that CYP17A1 is not only a classical steroidogenic enzyme but also a context-dependent metabolic modulator. In obesity and other states of metabolic stress, aberrant CYP17A1 expression may promote excessive production of steroid intermediates such as 17-hydroxyprogesterone, thereby enhancing glucocorticoid receptor signaling, increasing hepatic gluconeogenesis, and worsening insulin resistance. In parallel, altered CYP17A1 activity may contribute to adipose tissue dysfunction, visceral fat accumulation, and hypertension, reinforcing its broader role in metabolic syndrome and type 2 diabetes [18,30,78].

Genetic studies further support the clinical relevance of CYP17A1 in metabolic disease. Polymorphisms within the CYP17A1 locus have been associated with type 2 diabetes susceptibility, glycemic traits, arterial stiffness, and other cardiometabolic phenotypes, suggesting that inter-individual variation in CYP17A1 activity may shape metabolic risk. These findings also indicate that CYP17A1 may function as a shared genetic node linking endocrine and vascular complications of metabolic disease [79,84]. However, it is important to note that many of these findings are preclinical or murine-based, and direct human data connecting tissue-specific CYP17A1 activity, steroid intermediates and metabolic outcomes remain limited. Hence, further investigation is necessary to validate the scope of CYP17A1 involvement in metabolic disorders and if this can then be utilized as a therapeutic target for T2DM.

Additionally, therapeutic targeting of CYP17A1 is complex. Although CYP17A1 inhibition can improve glucose homeostasis and reduce steroid-driven metabolic dysfunction in experimental models, broad inhibition of this enzyme may also disrupt cortisol and sex steroid synthesis and trigger undesirable endocrine effects. Therefore, a more precise strategy may involve identifying upstream regulators and downstream effectors of CYP17A1, including glucocorticoid receptor signaling and tissue-specific steroidogenic pathways, rather than relying solely on complete enzyme inhibition [30,135,140].

As the prevalence of obesity, diabetes, and metabolic syndrome continues to rise globally, further studies are needed to clarify the tissue-specific roles of CYP17A1, the functional significance of its genetic variants, and the long-term metabolic consequences of modulating its activity. A deeper understanding of CYP17A1-mediated steroid metabolism may ultimately support the development of novel preventive and therapeutic strategies for metabolic disorders [78,79,135].

## Figures and Tables

**Table 1:** An overview of the role of CYP17A1 pathway dysregulation in metabolic disorders.

Title	Sample	Findings	Source
Dysregulation of cytochrome P450c17a as the cause of polycystic ovarian syndrome	*In vivo* PMOS and non-PMOS patients	The study demonstrated exaggerated 17-hydroxyprogesterone and androstenedione responses to GnRH agonist stimulation in women with (PMOS), suggesting hyperactivity of ovarian CYP17A1 rather than a steroidogenic block.	[74]
A polymorphism in the *CYP17A1* gene influences the therapeutic response to steroidogenesis inhibitors in Cushing’s syndrome	*In vivo* human adrenal NCI-H295R cells	In patients with Cushing’s syndrome treated with ketoconazole and/or metyrapone, the CYP17A1 *rs6163* polymorphism significantly influenced therapeutic response, with CC homozygotes more likely to achieve normalization of urinary free cortisol and doing so in a shorter time frame compared with AA/AC carriers.	[75]
Polycystic ovaries and premature male pattern baldness are associated with one allele of the steroid metabolism gene CYP17	*In vivo* human genetic analysis	In women with polycystic ovaries and premature male pattern baldness, a promoter polymorphism in CYP17 (–34 T→C, A2 allele) was significantly associated with the affected phenotype, suggesting increased androgen biosynthesis in carriers; however, linkage analysis demonstrated that this variant does not co-segregate with disease and therefore functions as a modifier rather than the primary causative mutation.	[76]
CYP17A1 deficient XY mice display susceptibility to atherosclerosis, altered lipidomic profile and atypical sex development	*In vivo* CYP17A1 and ApoE *KO* mice	When Cyp17a1-deficient mice were crossed with Apoe knockout mice, XY double-knockout animals developed significantly greater atherosclerotic burden and altered lipid profile compared with Apoe controls	[77]
Obesity-induced excess of 17-hydroxyprogesterone promotes hyperglycemia through activation of glucocorticoid receptor	*In vivo* and *in vitro* human HepG2 cells Liver biopsy Mice	Obesity upregulates hepatic CYP17A1, leading to increased 17-hydroxyprogesterone (17-OHP), which directly activates the glucocorticoid receptor and promotes hyperglycemia and insulin resistance.	[30]
Mutated CYP17A1 promotes atherosclerosis and early-onset coronary artery disease	*In vivo* Cyp17a1 *KO* mice	The Cyp17a1 mutation segregated with metabolic syndrome in affected families and, consistent with this, Cyp17a1-deficient mice developed hypertension, hyperglycemia, central obesity, fatty liver, and increased atherosclerosis.	[78]
A Study on the Association Between Polymorphisms in the CYP17A1 Gene Region and Type 2 Diabetes Mellitus in Han Chinese	*In vivo* genetic analysis of diabetic subjects and controls	CYP17A1 polymorphisms rs1004467, rs17115149, and rs12413409 were significantly associated with increased risk of type 2 diabetes and higher plasma glucose levels.	[79]
CYP17A1 Maintains the Survival of Glioblastomas by Regulating SAR1-Mediated Endoplasmic Reticulum Health and Redox Homeostasis	*In vivo* mice xenografted with primary human glioblastoma cells	Cyp17a1 maintains glioblastoma survival through SAR1-dependent regulation of ER integrity and redox homeostasis, and pharmacologic inhibition with abiraterone disrupts this pathway reducing growth *in vitro*.	[80]
Abiraterone and Increased Survival in Metastatic Prostate Cancer	Humans with metastatic castration-resistant prostate cancer	Abiraterone plus prednisone significantly improved overall survival in men with metastatic castration-resistant prostate cancer previously treated with docetaxel.	[81]
Shift from visceral to subcutaneous adipose tissue in *Cyp17a1*-knockout rats prevents the progression of metabolic syndrome	*In vivo* sample of adipose tissue Cyp17a1 KO in rats using CRISPR-Cas9	Deletion of Cyp17a1 induced obesity characterized by a preferential expansion and enhanced adipogenic activity of subcutaneous adipose tissue, yet did not result in insulin resistance, dyslipidemia, or hypertension, thereby demonstrating a metabolically healthy obesity phenotype driven by depot-specific metabolic redistribution.	[18]
Association of CYP17A1 Genetic Polymorphisms and Susceptibility to Essential Hypertension in the Southwest Han Chinese Population	Human genetic analysis of eight SNPs of CYP17A1	CYP17A1 polymorphisms *rs11191548* and *rs4919687* were significantly associated with essential hypertension, with *rs4919687* predicted to affect splicing and transcription factor binding, and specific multi-SNP haplotypes conferring increased susceptibility.	[82]
Semaglutide Alleviates Ovary Inflammation via the AMPK/SIRT1/NF-κB Signaling Pathway in Polycystic Ovary Syndrome Mice	*In vivo* study of PMOS mice with IHC of ovaries	In PMOS mice, Cyp17a1 is overexpressed in ovarian tissue, and semaglutide treatment significantly downregulates Cyp17a1 expression, thereby helping reduce testosterone levels and improve hormonal imbalance.	[83]
Genetic variation in CYP17A1 is associated with arterial stiffness in diabetic subjects	*In vivo* analysis of 52 SNPs in humans	Among 52 hypertension-associated SNPs, only a CYP17A1 variant remained significant after correction and was strongly associated with increased augmentation index in normotensive diabetic individuals, suggesting a link between CYP17A1 and early arterial stiffness and vascular dysfunction.	[84]

## Data Availability

No new data were created or analyzed in this study. Data sharing does not apply to this article.
